# A Thorough Examination of Peltophorum pterocarpum Phytochemicals in Network Pharmacology-Based Management of Acne Vulgaris

**DOI:** 10.7759/cureus.68159

**Published:** 2024-08-29

**Authors:** Aswathi K Biju, Nisha B., Rajeshkumar Shanmugam

**Affiliations:** 1 Nanobiomedicine Lab, Centre for Global Health Research, Saveetha Medical College and Hospitals, Saveetha Institute of Medical and Technical Sciences, Saveetha University, Chennai, IND; 2 Community Medicine, Saveetha Medical College and Hospitals, Saveetha Institute of Medical and Technical Sciences, Saveetha University, Chennai, IND

**Keywords:** disease, plant, potential genes, p. pterocarpum, phytochemicals, multi-targets, acne

## Abstract

Introduction

Acne vulgaris is a common skin problem caused by inflammation of the sebaceous glands and hair follicles as a result of hormonal fluctuations, bacteria, and overproduction of oil. The plant *Peltophorum pterocarpum* (*P. pterocarpum*) has been investigated for possible medical uses. Its anti-inflammatory, antibacterial, and antioxidant qualities are well recognised, and they may be applied to treat several diseases. This study investigates the plant's phytochemicals for their effectiveness in treating acne.

Methods

The Indian Medicinal Plants, Phytochemistry and Therapeutics (IMPPAT) database was utilised to extract the phytochemicals from *P. pterocarpum*. The absorption, distribution, metabolism, and excretion (ADME) analysis was conducted using the online tool SwissADME. The SwissTargetPrediction tool determines the potential targets of these phytochemicals. Targets for acne have been identified using the Open Targets Platform database. The common targets of *P. pterocarpum* and acne were identified using the Venn diagram drawing tool Venny 2.1.0, and a protein-protein interaction (PPI) network was built using the Search Tool for the Retrieval of Interacting Genes/Proteins (STRING) database. Following that, hub genes were identified by Cytoscape 3.10.2. The web tool ShinyGO 0.80 has enabled simpler evaluation of enrichment analysis for these hub genes.

Results

Five genes were shown to be key targets because they are directly engaged in the relaxin signalling pathway by pathway analysis: epidermal growth factor receptor (EGFR) and various matrix metalloproteinases (MMPs) (MMP1, MMP2, MMP9, and MMP13). The phytochemicals found in *P. pterocarpum*, including quercetin, rhamnetin, hirsutidin, and (+)-leucocyanidin, target these key genes.

Conclusion

This study highlights the potential of *P. pterocarpum* as a multi-target therapeutic agent for acne vulgaris. By targeting key genes in the relaxin signalling pathway, the phytochemicals from *P. pterocarpum* present a promising approach for acne management.

## Introduction

Acne vulgaris is a persistent skin condition that affects the sebaceous glands and hair follicles. It is caused by several factors, including skin microorganisms [1]. A wide variety of microorganisms make up the skin microbiome within the follicle. *Propionibacterium acnes* and *Malassezia* spp. are associated with the development of acne due to their impact on sebum production, inflammatory response, and formation of comedones [2]. Although it affects people of all racial and ethnic backgrounds, acne vulgaris, one of the most common disorders in dermatology, presents differently in each skin type [3]. Severe acne can have a substantial negative impact on a person's psychological health and quality of life [4]. Several factors have been identified as contributing to the production of acne, including air pollution, aggressive face creams, the skin and gut flora, genetic predisposition, hormones, psychological stress, and certain medications. Diet is also known to directly correlate with the transcription of genes linked to sebaceous gland function, bacterial growth, and inflammation, all of which promote the development of the disease [5]. The multiple clinical varieties of acne are distinguished by the physical traits and intensity of the lesions. These include severe inflammatory acne (including nodular and clustered acne) with nodules or cysts; papulopustular acne, characterised by the presence of inflammatory lesions (papules and/or pustules); comedonal acne, which is primarily composed of closed and/or open comedones; fulminant acne; and purulent acne [6]. The appropriate therapy for acne may be chosen by analysing its severity. Early action in the treatment of acne is recommended to reduce the chance of scarring and negative psychological consequences [7]. The use of medicinal plants has become a focal point of research due to their potential efficacy and safety for acne treatment.

*Peltophorum pterocarpum* (*P. pterocarpum*), commonly known as yellow flamboyant, is a well-known ornamental tree that belongs to the Fabaceae family and is native to tropical south-eastern Asia [8]. Various parts of this tree are utilised for treating a variety of diseases, including skin problems, ringworm, constipation, stomatitis, and insomnia. Its floral extract is also believed to treat insomnia. Its bark is used as an eye lotion, an embrocation for sores and aches, and a medication for diarrhoea [9]. Traditional healers use a decoction made from the leaves to treat skin diseases. A stem infusion of *P. pterocarpum* is used for gargles, toothpowder, and muscle aches. The flowers can be used as a lotion for muscle aches, eye problems, and sores, or as an astringent to treat or reduce intestinal diseases, bruises, sprains, childbirth pain, and swelling [10].

Network pharmacology is a rapidly developing field in drug research and development that combines information technology with systematic medicine. It is an integrated in silico method, which aims to create a "protein-phytochemical/disease-gene" network to uncover the mechanisms behind the complementary therapeutic effects of traditional drugs [11]. In this study, the multi-target pharmacological effect of *P. pterocarpum* against acne was thoroughly clarified through the application of the network pharmacology technique. The study explored multi-target biological functions and associated pathways to determine the effectiveness of *P. pterocarpum* against acne. Based on an extensive understanding of the potential therapeutic effects of *P. pterocarpum*, the findings suggest the utilisation of this plant as a multitargeted option for treating acne.

## Materials and methods

Phytochemical in *P. pterocarpum*

Phytochemicals from *P. pterocarpum* were sourced using the keyword "*Peltophorum pterocarpum*" in the Indian Medicinal Plants, Phytochemistry and Therapeutics (IMPPAT) database (IMPPAT - retrieved on June 28, 2024) [12,13]. Phytochemical chemical structures are represented using the standard approach known as Simplified Molecular Input Line Entry System (SMILES). PubChem was used to gather the SMILES of phytochemicals for target identification (PubChem - retrieved on June 28, 2024).

Absorption, distribution, metabolism, and excretion (ADME) screening

The SwissADME web tool (SwissADME - retrieved on June 28, 2024) was utilised to conduct a thorough evaluation of the ADME characteristics of the phytochemicals [14]. Because of their high gastrointestinal (GI) absorption and a threshold for oral bioavailability of 0.3 or more, *P. pterocarpum* phytochemicals were chosen for study.

Target identification of *P. pterocarpum*

An advanced online platform called SwissTargetPrediction is used to predict the biological targets of phytochemicals (SwissTargetPrediction - retrieved on June 28, 2024) [15]. The tool was updated with the canonical SMILES of phytochemicals that meet ADME requirements and identify targets based on a likelihood score of 0.1 or higher. These targets, which demonstrate tremendous potential, are then designated for thorough and extensive investigation.

Acne-associated target identification

The Open Targets Platform offers an abundance of data on predicted and annotated targets linked to human diseases (Open Targets Platform - retrieved on June 28, 2024) [16]. To find molecular targets related to acne, this database was used. A global score limit of 0.3 or higher was applied to screen targets. To identify similar targets between phytochemicals from *P. pterocarpum* and targets associated with acne, the Venn diagram application Venny 2.1.0 was utilised to identify overlapping targets (Venny 2.1.0 - retrieved on June 28, 2024) [17]. To further illustrate the connections between plants, phytochemicals, targets, and disease, a network was constructed using Cytoscape 3.10.2.

Building protein-protein interaction (PPI) network and identification of hub genes

The common targets were fed into the Search Tool for the Retrieval of Interacting Genes/Proteins, v 12.0 (STRING 12.0) tool to create the PPI network (STRING - retrieved on June 28, 2024) [18]. The species "Homo sapiens" was chosen, with a medium confidence level of 0.400; all other attributes were left unaltered. Subsequently, the PPI findings were imported into Cytoscape 3.10.2, which allowed for the integration of attribute data and the visualisation of intricate networks. The hub genes that are important in the complex PPI network were identified by applying the maximum clique centrality (MCC) approach of the CytoHubba plugin (Cytoscape - retrieved on June 28, 2024).

Functional enrichment analysis

ShinyGO 0.80, a graphical tool for the analysis of enrichment (ShinyGO 0.80 - retrieved on June 28, 2024), was used to conduct the functional enrichment analysis: Kyoto Encyclopaedia of Genes and Genomes (KEGG) and Gene Ontology (GO) studies on the hub genes [19]. The analysis focused exclusively on "Homo sapiens" as the accepted species. Lollipop plots were utilised to visualise the enriched GO keywords for cellular component (CC), biological process (BP), and molecular function (MF), with a false discovery rate (FDR) level set at 0.05. Lollipop plots were produced to show the fold enrichment, gene counts from the GO analysis, and -log10FDR in relation to the distribution of genes linked to each process. Furthermore, KEGG pathway analysis was used to identify pathways that were relevant to the selected targets, in accordance with the overall number of genes linked to each pathway.

## Results

Target identification

**Figure 1:**
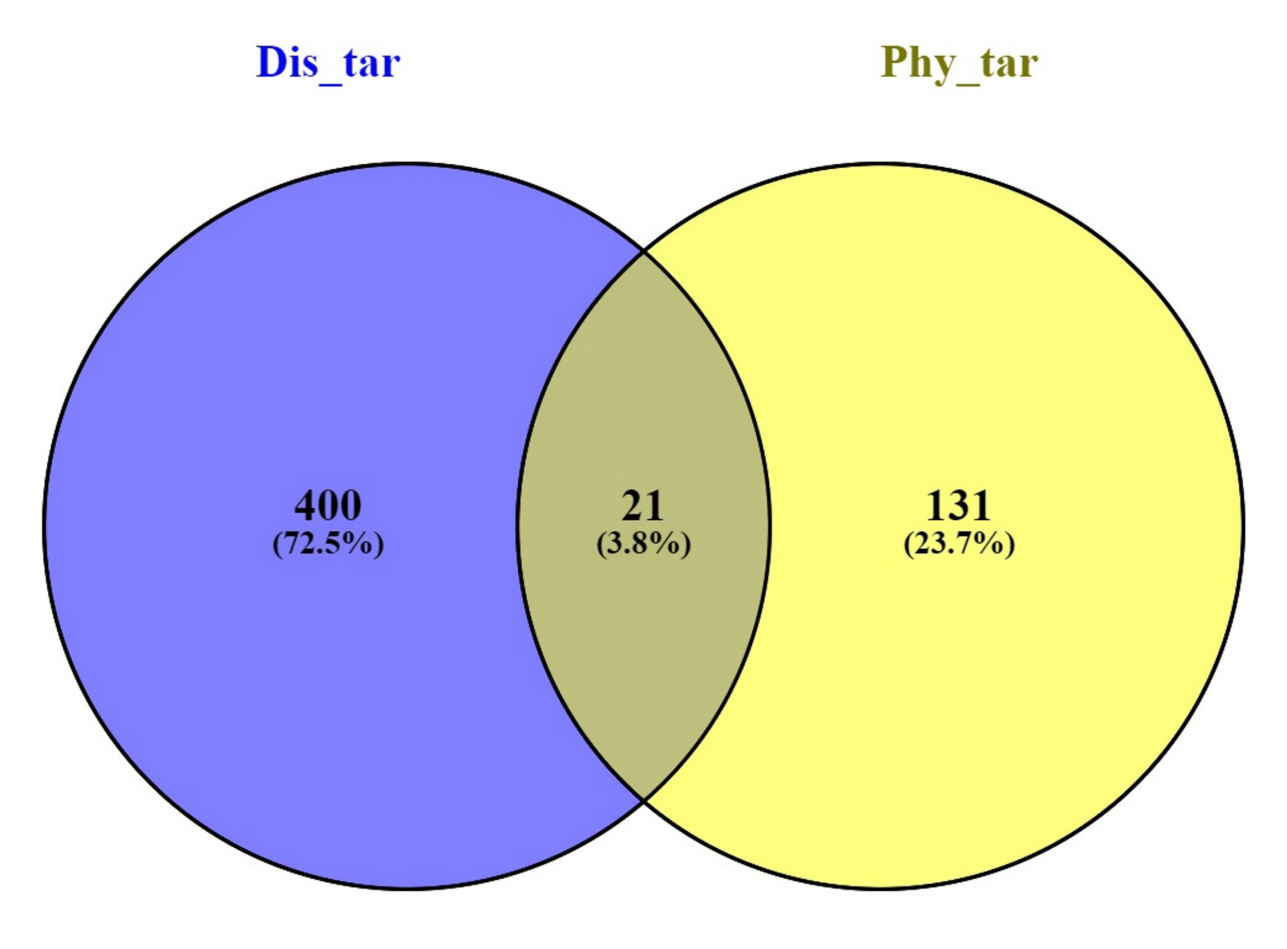
Venn diagram demonstrating shared genes between P. pterocarpum (phytochemical targets as Phy_tar) and acne (disease targets as Dis_tar) Image credit: Aswathi K. Biju *P. pterocarpum*: *Peltophorum pterocarpum*

**Figure 2:**
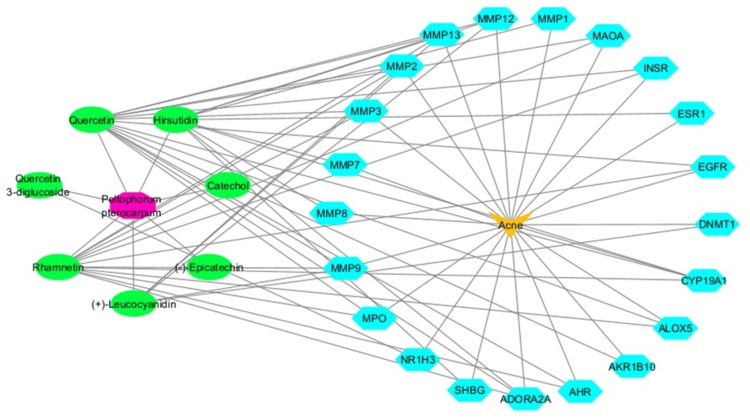
Plant-phytochemical-target-disease network *Peltophorum pterocarpum* is represented by rose colour nodes, plant phytochemicals by green colour nodes, common targets for both phytochemicals and disease by blue nodes, and disease is represented by yellow colour nodes. Image credit: Aswathi K. Biju MMP: Matrix metalloproteinase; MAOA: Monoamine oxidase A; INSR: Insulin receptor; ESR1: Estrogen receptor 1; EGFR: Epidermal growth factor receptor; DNMT1: DNA methyltransferase 1; CYP19A1: Cytochrome P450 family 19 subfamily A member 1; ALOX5: Arachidonate 5-lipoxygenase; AKR1B10: Aldo-keto reductase family 1 member B10; MPO: Myeloperoxidase; NR1H3: Nuclear receptor subfamily 1 group H member 3; SHBG: Sex hormone-binding globulin; AHR: Aryl hydrocarbon receptor; ADORA2A: Adenosine A2A receptor

Using the IMPPAT database, 15 phytochemicals were found in *P. pterocarpum*; seven of these compounds satisfied the requirements for ADME screening (Table 1). The SwissTargetPrediction online tool was then utilised to submit the canonical SMILES of these seven compounds for target prediction. Using this approach, 152 targets were obtained with a probability value of 0.1 or higher. Furthermore, 421 targets associated with acne, which had a global score of 0.01 or higher, were retrieved from the Open Targets Platform. Using Venny 2.1.0, the phytochemical-targeted genes of *P. pterocarpum* were cross-referenced with disease-specific genes for acne to find shared genes. Based on the results from the Venn diagram, 21 common targets were identified and are the subjects of additional research (Figure 1). Cytoscape 3.10.2 was used to generate a degree-sorted circular arrangement, which allowed for a methodical visualisation of the plant-phytochemical-target-disease network (Figure 2).

**Table 1:** ADME (absorption, distribution, metabolism, and excretion) qualified phytochemicals of P. pterocarpum *P. pterocarpum*: *Peltophorum pterocarpum*

Sl No	*P. pterocarpum*’s phytochemicals	Molecular weight	Gastrointestinal (GI) absorption	Bioavailability
1	Catechol	110.11	High	0.55
2	Quercetin	302.24	High	0.55
3	(+)-Leucocyanidin	306.27	High	0.55
4	(-)-Epicatechin	290.27	High	0.55
5	Hirsutidin	345.32	High	0.55
6	Quercetin 3-diglucoside	345.32	High	0.55
7	Rhamnetin	316.26	High	0.55

Construction of PPI network and screening of hub gene

**Figure 3:**
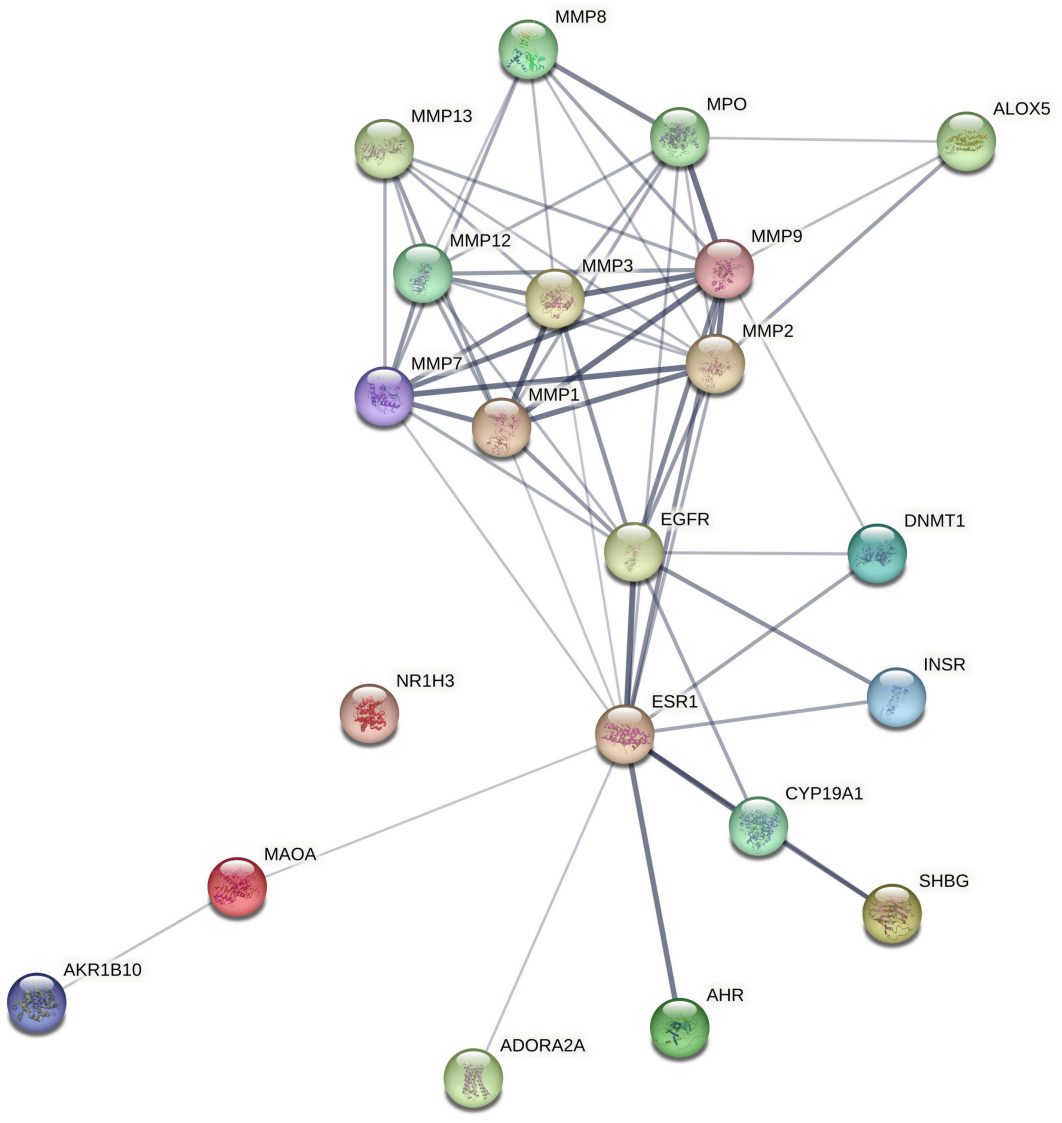
Protein-protein interactions network of commonly targeted genes of Peltophorum pterocarpum and acne Image credit: Aswathi K. Biju MMP: Matrix metalloproteinase; MAOA: Monoamine oxidase A; INSR: Insulin receptor; ESR1: Estrogen receptor 1; EGFR: Epidermal growth factor receptor; DNMT1: DNA methyltransferase 1; CYP19A1: Cytochrome P450 family 19 subfamily A member 1; ALOX5: Arachidonate 5-lipoxygenase; AKR1B10: Aldo-keto reductase family 1 member B10; MPO: Myeloperoxidase; NR1H3: Nuclear receptor subfamily 1 group H member 3; SHBG: Sex hormone-binding globulin; AHR: Aryl hydrocarbon receptor; ADORA2A: Adenosine A2A receptor

**Figure 4:**
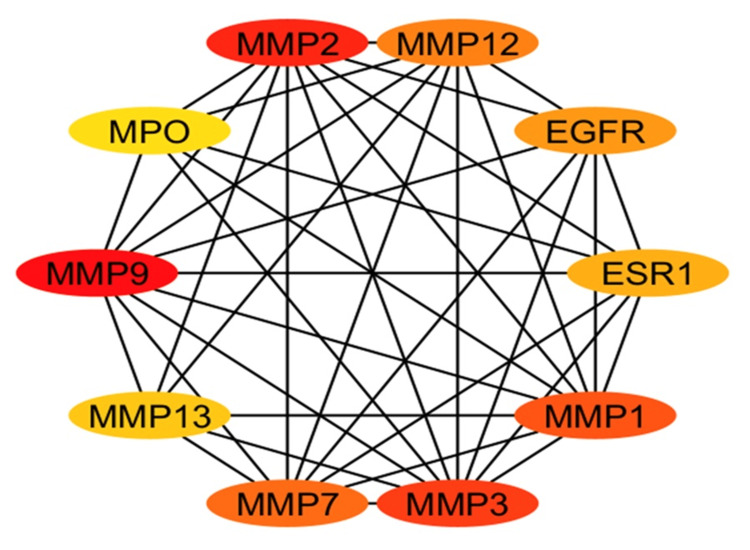
Top 10 hub genes derived from protein-protein interaction network of common targets Image credit: Aswathi K. Biju MPO: Myeloperoxidase; MMP: Matrix metalloproteinase; EGFR: Epidermal growth factor receptor; ESR 1: Estrogen receptor 1

Employing the STRING database, a PPI network with an enrichment p-value of less than 1.0 × 10^-16^ was built for the 53 common targets of *P. pterocarpum* and acne. The PPI network shows 21 nodes (genes) and 61 edges (interactions) between the genes, which has an average node degree of 5.81. The PPI network's edge thickness reveals the degree of data support (Figure 3). Following that, the PPI network was analysed using the CytoHubba plugin, and the MCC technique was used to determine the top 10 most important genes (Figure 4). Hub genes play a critical role in determining the cause of disease because of their high degrees of interaction. The top 10 hub genes identified are matrix metalloproteinases (MMPs) MMP9, MMP2, MMP3, MMP1, MMP7, MMP12, epidermal growth factor receptor (EGFR), estrogen receptor 1 (ESR1), MMP13, and myeloperoxidase (MPO).

GO studies and KEGG pathway enrichment analysis

**Figure 5:**
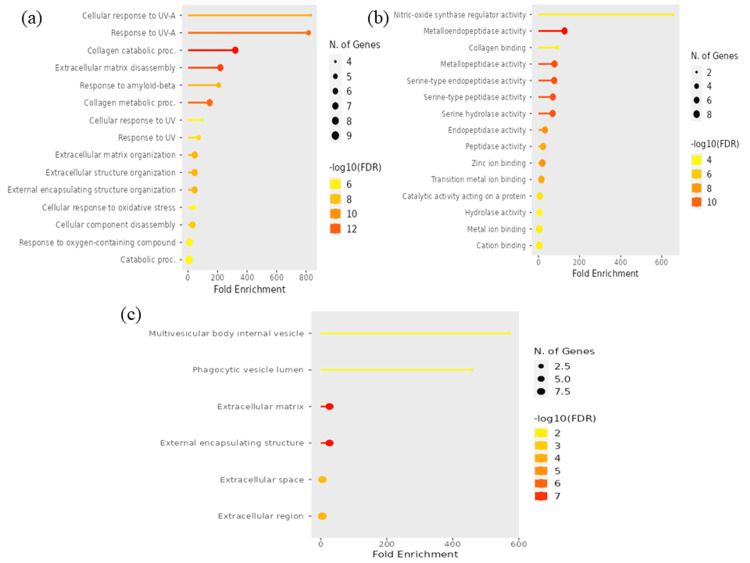
Lollipop plots demonstrating the gene ontology of the targeted genes These plots display the part of gene ontology enrichment analysis: biological process (a), molecular function (b), and cellular component (c) of targeted genes. Image credit: Aswathi K. Biju FDR: False discovery rate

**Figure 6:**
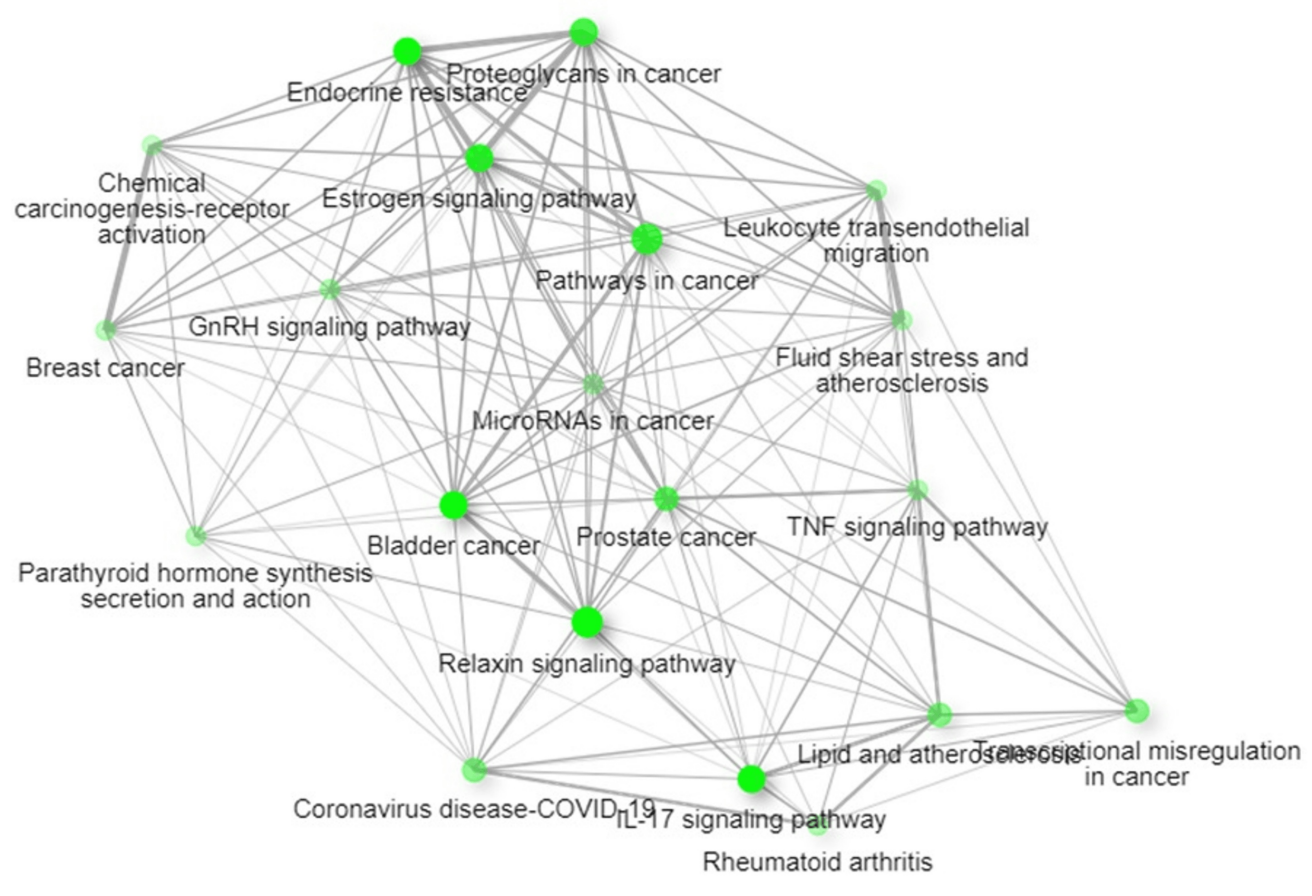
Topmost 20 KEGG pathway network of the hub genes Image credit: Aswathi K. Biju KEGG: Kyoto encyclopaedia of genes and genomes; GnRH: Gonadotropin-releasing hormone; TNF: Tumor necrosis factor; IL-17: Interleukin-17

**Figure 7:**
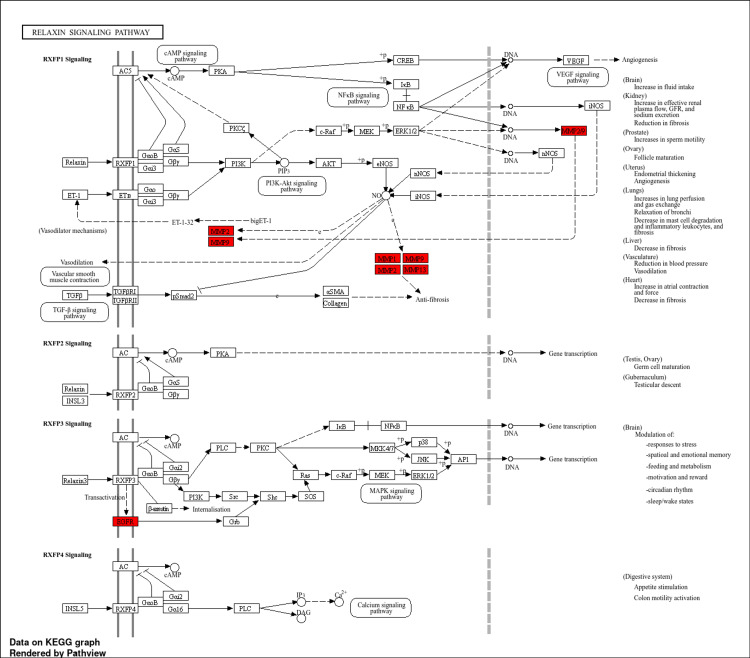
Relaxin signalling pathway, demonstrating the core targets in red colour was generated using ShinyGO 0.80 Image credit: Aswathi K. Biju RXFP: Relaxin/insulin-like family peptide; Gαs: G protein alpha subunit; Gβγ: G protein beta-gamma subunit; AC: Adenylyl cyclase; cAMP: Cyclic adenosine monophosphate; PKA/C: Protein kinase A/C; CREB: cAMP response element-binding protein; PI3K: Phosphoinositide 3-kinase; MEK: Mitogen-activated protein kinase/extracellular signal-regulated kinase; ERK1/2: Extracellular signal-regulated kinase 1/2; p38 MAPK: p38 mitogen-activated protein kinase; eNOS: Endothelial nitric oxide synthase; nNOS: Neuronal nitric oxide synthase; iNOS: Inducible nitric oxide synthase; TGF-β: Transforming growth factor beta; Smad2: Mothers against decapentaplegic homolog 2; αSMA: Alpha-smooth muscle actin; MMP: Matrix metalloproteinase; ET-1: Endothelin-1; ETB: Endothelin receptor type B; Akt: Protein kinase B; VEGF: Vascular endothelial growth factor; SOX9: SRY-box transcription factor 9; MAPK: Mitogen-activated protein kinase; PLC: Phospholipase C; DAG: Diacylglycerol; PIK: Phosphoinositide kinase

To obtain greater insight into the roles of the 10 hub genes, GO and KEGG investigations were performed. The GO terms covering the BP, MF, and CC of the targeted genes were plotted on a lollipop plot (Figure 5). The process names are listed on the Y-axis, while fold enrichment can be observed on the X-axis. The -log10FDR and gene count can be observed by the size and colour of each lollipop, respectively. Analysis of the targeted genes' BPs showed that they are mostly engaged in the cellular response to UV-A and response to UV-A. These genes were discovered to be connected to nitric-oxide synthase regulator activity in terms of MF. The targeted genes were shown to be involved in the multivesicular body internal vesicle and phagocytic vesicle lumen by CC analysis. Using an FDR threshold of 0.05, KEGG pathway enrichment analysis revealed the top 20 biological pathways associated with the hub genes. The relaxin signalling pathway and cancer pathway show the highest gene counts among them, and the hub genes were significantly implicated in these pathways. The total number of genes linked to each pathway is represented by the size of the pathway nodes in the pathway network. The thickness of the edges indicates the number of overlapping genes (Figure 6). Five genes have been identified as core targets that have a direct role in the relaxin signalling pathway: EGFR, MMP1, MMP2, MMP9, and MMP13. Within the relaxin signalling pathway, these core targeted genes are indicated in red (Figure 7). These vital genes have been found to be targeted by four phytochemicals derived from *P. pterocarpum*: quercetin, rhamnetin, hirsutidin, and (+)-leucocyanidin.

## Discussion

In this study, we used network pharmacology to examine the multi-target pharmacological action of *P. pterocarpum* against acne. Among the 21 common targets of *P. pterocarpum* and acne, five targets - EGFR, MMP1, MMP2, MMP9, and MMP13 - were selected as the core targets because of their direct involvement in the relaxin signalling pathway. Relaxin is a peptide hormone that mediates a range of physiological processes across various tissues, such as cardiovascular, renal, hepatic, reproductive, and particularly connective tissues [20]. It does this by activating the G protein-coupled receptor (GPCR), relaxin family peptide receptor 1 (RXFP1). Relaxin is an important regulator of connective tissue integrity and remodelling due to its capacity to activate multiple intracellular signalling pathways, including mitogen-activated protein kinases (MAPKs), cyclic adenosine monophosphate (cAMP), Akt (protein kinase B), nitric oxide (NO), protein kinase C (PKC), cyclic guanosine monophosphate (cGMP), and phosphatidylinositol-4,5-bisphosphate 3-kinase (PI3K) [20,21]. Relaxin plays a crucial role in connective tissue dysplasia (CTD), a condition marked by abnormal connective tissue development. Its impact on collagen production and tissue remodelling makes it particularly significant in understanding and potentially managing CTD. Acne is frequently associated with altered tissue architecture and inflammation, making recurrences more prevalent in individuals with CTD. These conditions can also have a more severe course. More severe acne in these individuals points to a possible disturbance in regular connective tissue healing and homeostasis, which may be related to dysregulated or inadequate relaxin signalling [22,23].

With the regulatory roles that relaxin plays on connective tissues, it is conceivable that improving or modifying relaxin signalling might be therapeutically beneficial for acne sufferers. Focusing on pathways related to tissue remodelling, cellular signalling, and inflammation - such as those regulated by MAPKs, cAMP, NO, and PI3K/Akt - relaxin may help these individuals regain normal tissue function and experience less severe acne [20,22]. The relaxin signalling pathway has emerged as a significant player in acne management. Previous research has identified this pathway as being involved in acne and sebum balance. Our findings corroborate these results, further highlighting the pathway’s relevance in acne treatment. Specifically, the modulation of EGFR and various MMPs (MMP1, MMP2, MMP9, and MMP13) through the relaxin signalling pathway appears to be a pivotal mechanism by which *P. pterocarpum* exerts its anti-acne effects [24]. EGFR is extremely important for the development, survival, and differentiation of skin and GI cells. Studies conducted in preclinical settings have demonstrated that inhibiting EGFR results in abnormalities and functional impairments of the GI tract and complexion. Skin toxicity is hence one of the main side effects of anti-EGFR treatment. The suppression of EGFR in tissues, especially in the skin and GI tract, is directly related to the risk profile of anti-EGFR treatments. Anti-EGFR therapy’s main side effect is skin toxicity, mostly in the form of a typical acneiform rash. In addition to rash, skin toxicity can also manifest as dry skin, especially after using TKIs. The rash can also appear maculopapular or pustular [25]. Various MMP1 and MMP2 expression levels may play a role in the formation of different types of acne lesions [26]. Increased MMP9 levels are correlated with both the severity of acne and the size of inflammatory lesions, making it a potential biomarker for identifying the best course of therapy for acne [27]. MMP13, which is released by keratinocytes and found in facial sebum, was shown to be significantly reduced after isotretinoin therapy. This finding suggests that MMP13 plays a role in the development of acne and that reducing it may enhance the effectiveness of the treatment [28]. This research implies that MMPs and EGFR contribute to the development of acne and that lowering them may improve the effectiveness of therapy.

Limitations

The current study's results demonstrate the complementary multi-target and multi-pathway impact of *P. pterocarpum* in controlling acne, indicating that *P. pterocarpum*’s phytochemicals can influence several targets and thus interfere with many biological processes and signalling pathways. These results are predictions based on the data and have certain limitations. To elucidate the targets and signalling pathways of *P. pterocarpum* in controlling acne, further experimental studies will be conducted based on the findings of this investigation.

## Conclusions

The pharmacological effectiveness of *P. pterocarpum* as a comprehensive treatment option for acne has been clarified by this investigation. A thorough understanding of *P. pterocarpum*'s therapeutic potential has been made possible by combining ADME predictions, core target identification, and functional enrichment analysis. These studies are useful for functional annotation, pathway analysis, and integrating biological data to comprehend the disease mechanisms and pinpoint potential therapeutic targets. *P. pterocarpum* is shown to be a very promising and adaptable therapy for acne. The core targets - EGFR, MMP1, MMP2, MMP9, and MMP13 - that directly participate in the relaxin signalling pathway are useful in managing acne by targeting these core targets. According to the research, *P. pterocarpum* holds great promise for treating acne. To confirm its therapeutic efficacy, safety profile, and potential advantages as a medicinal plant, further experimental study is required.
